# Temporal metagenomic characterization of microbial community structure and nitrogen modification genes within an activated sludge bioreactor system

**DOI:** 10.1128/spectrum.02832-23

**Published:** 2023-11-29

**Authors:** Claire N. Freeman, Jennifer N. Russell, Chris K. Yost

**Affiliations:** 1 Department of Biology, University of Regina, Regina, Saskatchewan, Canada; 2 Department of Large Animal Clinical Sciences, University of Saskatchewan, Saskatoon, Saskatchewan, Canada; Connecticut Agricultural Experiment Station, New Haven, Connecticut, USA

**Keywords:** wastewater treatment, metagenomics, nitrogen cycle enzymes

## Abstract

The biological removal of nitrogen using natural microbial metabolic processes can be a valuable component of wastewater treatment that helps reduce downstream eutrophication of receiving water ecosystems. Biological nutrient removal (BNR) is a well-established component of wastewater treatment due to its recognized environmental benefits. The composition and diversity of these microbial communities are an important consideration, as disruptions to or instability in the microbial community can negatively impact N cycling and reduce treatment efficiency. To characterize the bacterial community and associated nitrogen cycling genes within a cold-acclimated BNR facility, metagenomic sequencing combined with a read-based quantification strategy and metagenomic assembled genome (MAG) generation was used on samples collected from a Canadian prairie wastewater treatment plant. Generally, this system had a high abundance of Proteobacteria and Actinobacteria throughout the year, including the genera *Thiomonas, Tetrasphaera, Afipia,* and *Hyphomicrobium*. Communities remained stable throughout the different bioreactors in this system, while diversity varied between sampling months, demonstrating seasonal effects on the population dynamics. Genes involved in the denitrification pathway were abundant and distributed widely across different MAGs, while genes involved in nitrification were absent. Additionally, these genes remained stable across all sampling months, suggesting that the efficacy and robustness of this system rely on more than the taxonomic composition of the microbial community.

**IMPORTANCE:**

Wastewater treatment plays an essential role in minimizing negative impacts on downstream aquatic environments. Microbial communities are known to play a vital role in the wastewater treatment process, particularly in the removal of nitrogen and phosphorus, which can be especially damaging to aquatic ecosystems. There is limited understanding of how these microbial communities may change in response to fluctuating temperatures or how seasonality may impact their ability to participate in the treatment process. The findings of this study indicate that the microbial communities of wastewater are relatively stable both compositionally and functionally across fluctuating temperatures.

## INTRODUCTION

Wastewater treatment plants (WWTPs) provide critical infrastructure to protect both public health and the integrity of aquatic ecosystems. Biological nutrient removal (BNR) is a process used in these systems to reduce the threat of eutrophication in receiving waters (1). In BNR facilities, wastewater is mixed with an “activated sludge” of microorganisms capable of transforming and removing nutrients through natural metabolic activity (2), combined with the manipulation of environmental conditions and resource availability to encourage specific metabolic processes. A primary objective of BNR is the removal of biologically available nitrogen, which is accomplished through the combination of the microbial processes of denitrification and nitrification. Many bacterial species are capable of reducing nitrate during cellular energy generation, generally in the absence of oxygen (3). In contrast, nitrification is a specialized chemoautotrophic process only known to occur in a few bacterial species in activated sludge and is one of the most fastidious processes to optimize within a wastewater treatment system (4).

A critical consideration in the optimization of BNR is temperature, as colder regions can experience reduced growth rates of important nitrifying bacteria (5). To combat this, regions that experience fluctuating temperatures often implement flexible treatment strategies to adapt to both warm and cold temperatures (6), which can include altering the volume holding capacity and flow rates through the treatment reactors (5) to increase the duration of contact between the WWTP resident microbial community and the wastewater to be treated. However, extensively reducing or extending the duration of treatment can lead to operational difficulties resulting in insufficiently treated water. For example, a shortened treatment duration may fail to stimulate the formation of stable bacterial flocs, leaving small, suspended particles in the effluent (7).

Operational changes in conjunction with fluctuating external temperatures can negatively impact the microbial community in activated sludge (8 – 10). It is important to understand how these communities change in response to temperature changes during optimal operation; therefore, this study aims to characterize the microbial community within activated sludge in a region subject to substantial temperature fluctuations and test the hypothesis that microbial communities in activated sludge remain stable throughout the year. The specific aims were to: (i) identify the most abundant species and quantify changes in the microbial abundance throughout the year, (ii) quantify microbial genes involved in nitrogen transformations, and (iii) derive information about the organisms involved in nitrogen transformations by detecting nitrogen metabolism genes in metagenome-assembled genomes (MAGs).

## MATERIALS AND METHODS

### WWTP overview

Samples were collected from the Regina Wastewater Treatment Plant (50.475997, −104.751725) in Saskatchewan, Canada, which services a population of ca. 233,000. The WWTP is operated by EPCOR, a commercial WWTP operator in partnership with the City of Regina. This WWTP processes approximately 150 mL of influent per day from domestic, commercial, and industrial sources. Influent undergoes primary treatment: grit removal, sedimentation, and surface skimming. Effluent from primary treatment is divided equally into three parts and flows into the three bioreactors (designated A, B, and C), where BNR occurs. Each bioreactor includes eight separated zones of varying oxygen availability that wastewater flows through sequentially.

The Regina WWTP experiences substantial ambient temperature fluctuations throughout the year. On average, the warmest month is July with an average ambient temperature of 18.9°C and the coldest month is December, with an average ambient temperature of −12.4°C 11. This results in an average temperature of 10°C–20°C in the mixed liquor in cold and warm seasons, respectively (Table S1).

### Sample collection and processing

Activated sludge samples were collected on 19 December 2017, 13 February 2018, 17 April 2018, and 6 July 2018. On these dates, the WWTP was performing within standard operational specifications and releasing effluent that conformed to permit regulations (K. Gallant, personal communication). These dates were selected to cover the range in seasonal temperatures and to capture the microbial diversity seen during normal plant operation. Fifteen milliliter samples were collected from zones 1, 2, 4, and 8 (pre-anoxic, anaerobic, anoxic, and aerobic, respectively). Sludge was collected from each of the three bioreactors running in parallel (A, B, and C). The samples were stored and frozen at −20°C at the University of Regina prior to DNA extraction. In terms of the temperature of the incoming untreated influent, April had the lowest value, while July had the highest (Table S1). Of the dates sampled, April had both the lowest nitrogen and phosphorus removal efficiencies (N: 91.89%; P: 69.58%).

### DNA extraction and metagenomic sequencing

Samples were briefly (<5 min) thawed in an ice bath and filtered through a 0.45 µm pore size mixed cellulose ester filter (MilliporeSigma, Burlington, MA, USA) in duplicate. The filter containing the microbial community was retained and placed directly into lysis tubes from the DNeasy Powersoil DNA extraction kit (QIAGEN, Inc., Hilden, Germany). The filters were briefly macerated with a sterile spatula and further DNA extractions were carried out according to the manufacturer’s instructions. DNA extracts were diluted to 50 ng/µL and shipped to Genome Quebec (Montréal, Québec) on dry ice for library preparation and DNA sequencing. Sequencing library preparation was completed with the TruSeq PCR free kit (Illumina, San Diego, USA), and the samples were sequenced on three lanes of an Illumina HiSeq 4000, generating paired-end, 150-bp reads.

### Read-based bioinformatic analysis

Raw sequencing reads with a 31-mer match to PhiX, the Illumina internal control, were removed via bbduk v36.2 (12). To estimate the sequencing coverage of the microbial community, Nonpareil v.3.3.3 was used (13). Sequence quality was assessed using FastQC v0.11.6 (14), and reads were quality trimmed using bbduk at Q20 with a minimum length of 100 bp. Quality-trimmed reads were mapped to the MetaPhlAn2 v2.9.20 database with bowtie2 v2.3.5 (15, 16) using the *--very-sensitive* flag. Taxonomic classifications and associated abundances for each sample were conducted using MetaPhlAn2 v2.9.20. Relative abundance data were used to create a heat map of the most abundant (≥1.0%) microbial genera using the R package vegan v2.5.5 (17) using the Bray-Curtis distance metric; these data were visualized using ggdendroplot v0.1.0, ggplot2 v3.4.1 (18), and viridis v0.6.2 (19). Finally, functional profiling of the metagenomic reads was completed with the HUMAnN2 pipeline v0.11.2 (20) using the UniRef90 database.

Counts at the genus level were used to calculate alpha diversity within each sample according to the Inverse Simpson metric using the R package vegan. A generalized linear model (GLM) was used to quantify the effect of sampling date, bioreactor zone, and bioreactor on sample diversity. The model included an interaction between the bioreactor and zone and assumed the response followed a gamma distribution, as diversity values were continuous and ranged from 0 to 10. Pairwise *post hoc* comparisons between the diversity of different months were estimated from the model using the R package emmeans v1.4.3 (21). Additionally, to determine the relationship between different members of the microbial community and months, bioreactors, and zones, genus count data generated by MetaPhlAn2 was Hellinger transformed (22) and used in redundancy analysis (RDA) with vegan.

### Assembly-based bioinformatic analysis

To generate MAGs from the activated sludge environment, reads from each month were co-assembled using MEGAHIT v1.2.8 (23) and binned using MetaBAT v1.12.1 (24). The quality of the resultant bins was assessed via CheckM v1.1.1, which uses the presence of clade-specific marker genes to calculate the percent completion and percent contamination of each bin (25). Only bins that passed the threshold of ≥90% complete and ≤10% contaminated were considered for additional analyses. Within each bin, genes related to denitrification and nitrification were detected via Prokka software suite v1.14.5 (26). The taxonomy of each MAG was determined via GTDB-Tk v2.3.2 (27).

## RESULTS

### DNA sequencing

Sequencing yielded an average of 19.3 ± 2.2 million reads per sample for a total of 227 Gb of data (Table S2). After filtering and trimming the sequences, an average of 83% of reads remained. The Nonpareil analysis estimated that between 50% and 70% of the community was sequenced (Fig. S1).

### Taxonomic composition of activated sludge

**Fig 1:**
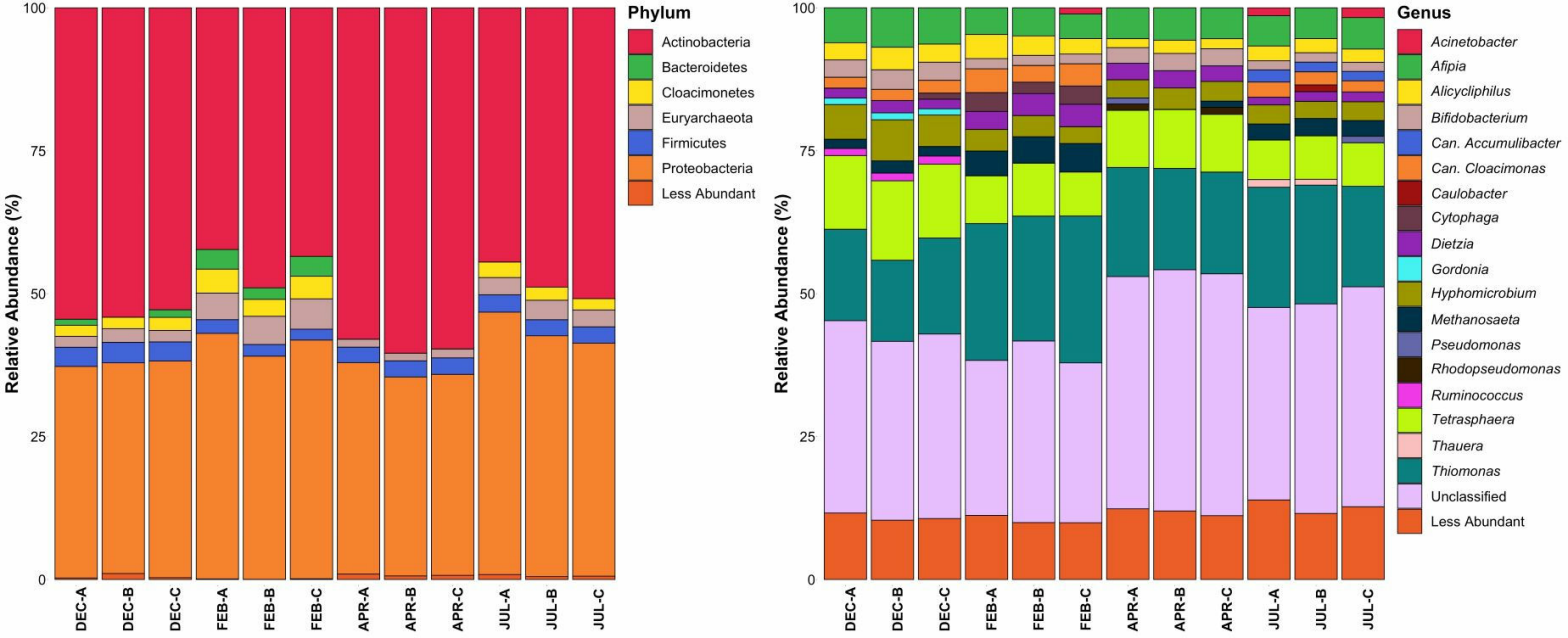
A summary of the phyla and genera that were present at ≥1.0% relative abundance. Count data were generated via MetaPhlAn2, and samples from the same date and bioreactor were averaged. Organisms that were <1.0% were summed and reclassified as “Less Abundant.” For the genera data, “*Can*.” refers to *Candidatus*, and “Unclassified” represents an unclassified genus belonging to the Dermatophilaceae family.

**Fig 2:**
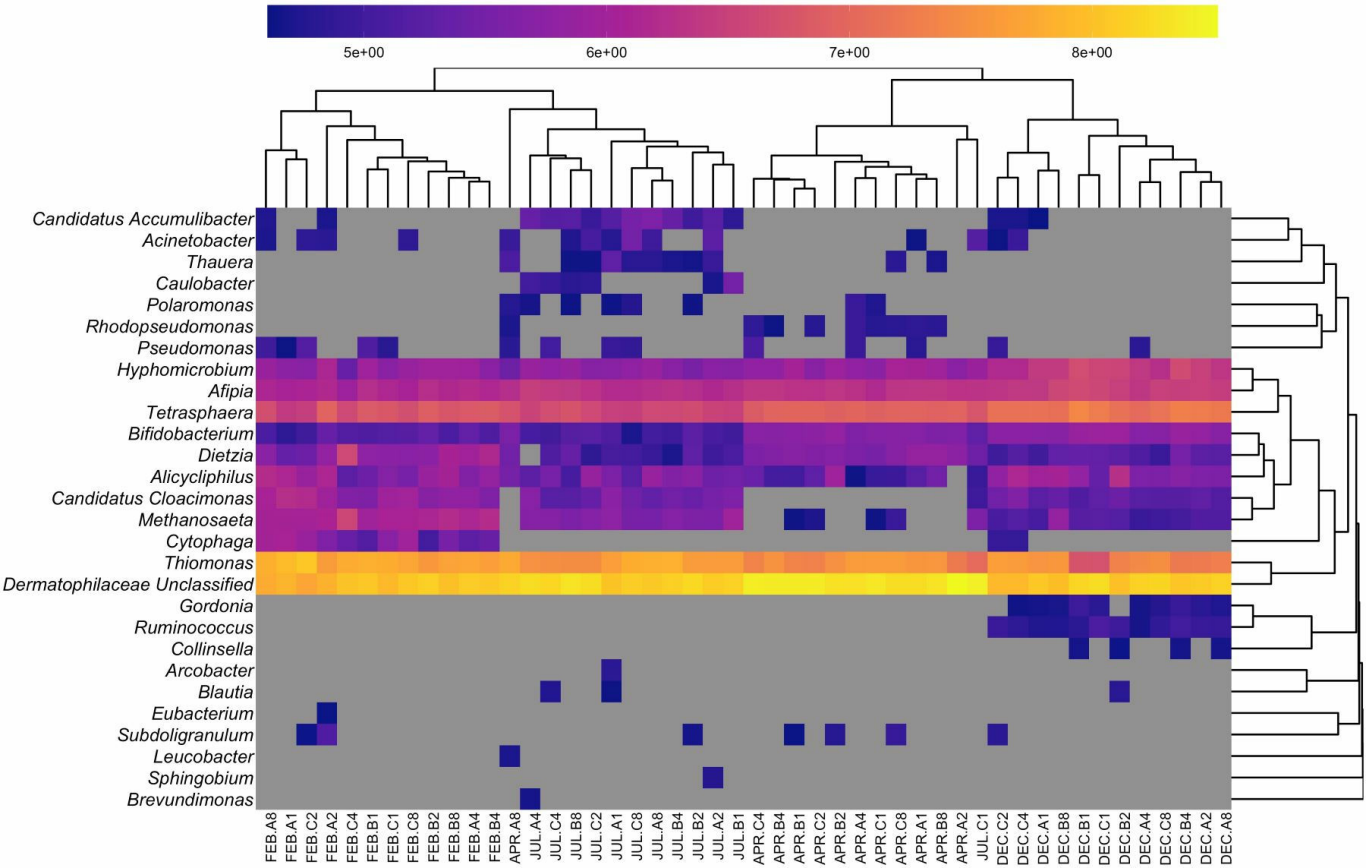
Heatmap and dendrogram of organisms with relative abundances greater than 1%. The abundance of the bacterial genera across all samples was determined via MetaPhlAn2. Dendrograms were generated using Bray-Curtis as the distance measure.

Microbial populations in the activated sludge were dominated by phyla Actinobacteria and Proteobacteria (Fig. 1). On average, Actinobacteria accounted for approximately 51% of the microbial community, while Proteobacteria accounted for 38%, although these proportions varied between months. Of the microbial community that could be classified at the genus level, the dominant organisms were *Thiomonas*, *Tetrasphaera*, *Afipia*, *Hyphomicrobium*, and *Dietzia*. At the genus level, differences between samples were most apparent between months rather than bioreactor or sampling locations (Fig. 2). Select genera have relatively consistent abundances throughout the year and do not fluctuate in response to seasonality (e.g., *Polaromonas*, *Collinsella*, and *Alicyphilus*), while others have pronounced differences between months or are absent altogether in certain months (e.g., *Candidatus Accumulibacter*, *Cytophaga,* and *Gordonia*).

### Genetic content

**Fig 3:**
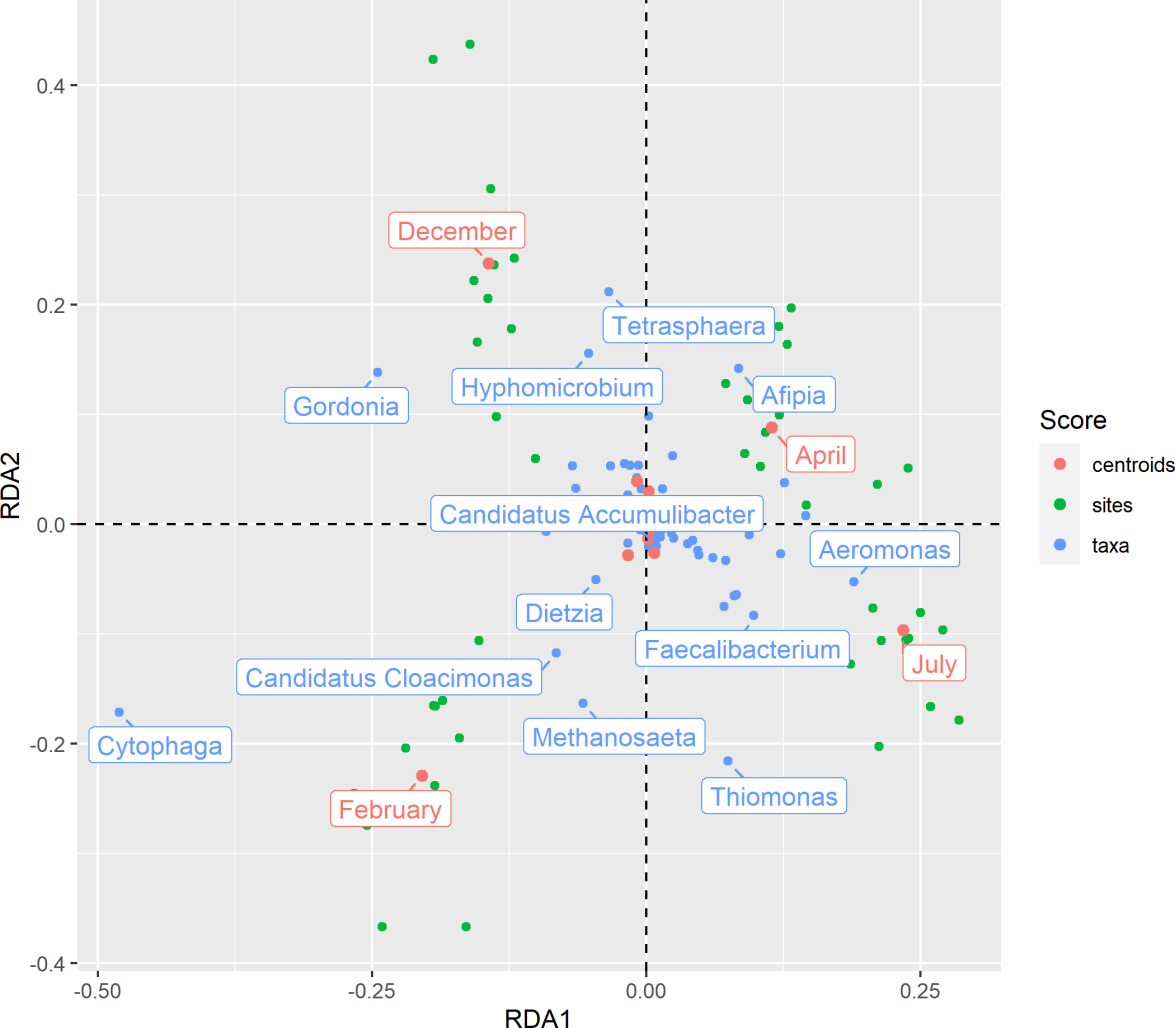
Abundances of enzymes involved in denitrification and nitrification in the Regina WWTP BNR system. Genes involved in nitrification and denitrification were detected and quantified using the command line tool, HUMAnN2. Each enzyme is present in similar abundances throughout the year with the exception of ammonia monooxygenase.

Across all samples, HUMAnN2 detected a total of 905,744 different UniRef hits, mapping to 130,600 different clusters of orthologous groups. To assess the genetic content of this microbial community in the context of nitrogen removal, genes involved in denitrification and nitrification were analyzed. Genes from various pathways in the nitrogen cycle were detected including denitrification, nitrogen fixation, assimilative and dissimilatory nitrogen reduction, and ammonia oxidation. All six enzymes necessary for complete nitrification and denitrification were present in varying abundances (Fig. 3). On average, denitrification genes were present in higher abundance (15 copies per million reads) than nitrification genes (<1 copy per million reads).

### Metagenome-assembled genomes

Metagenomic binning generated 74 MAGs that passed the threshold of ≥90% complete and ≤10% contaminated (Table S3). The majority of bins were from Domain Bacteria, but a single bin found only in February samples was classified as Archaea (bin.1; *Methanothrix soehngenii*). Of the 74 quality MAGs that could be classified at the phylum level, the majority were classified as Proteobacteria (*n* = 26), Bacteroidetes (*n* = 18), and Actinobacteria (*n* = 8). The majority of MAGs were only present in 1 month, although eight were present in all four sampling dates. Nitrate reductase and nitrite reductase were found in 42 and 8 MAGs, respectively. Genes encoding enzymes for nitrification and other genes involved in denitrification were not detected. Only three MAGs included both nitrate and nitrite reductase genes.

### Changes in the microbial community

The results of the GLM indicated that the differences between samples were best explained by the date the samples were collected. The diversity in this environment was found to be highest in December and pairwise comparisons between all other months showed insignificant differences (Table S4).

**Fig 4:**
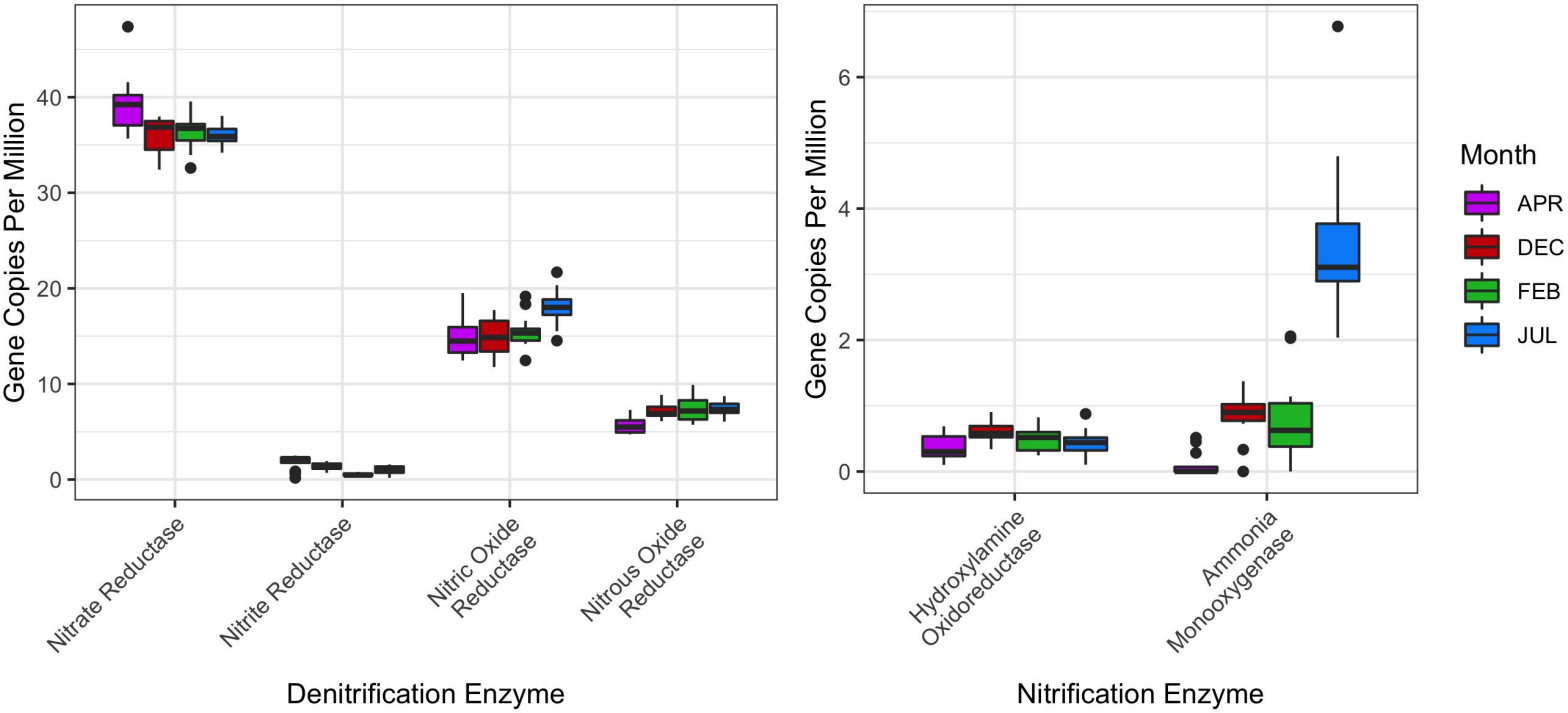
Redundancy analysis of the microbial communities in the Regina WWTP BNR system. The abundance of the bacterial genera across all samples was determined using the tool, MetaPhlAn2 and used in redundancy analysis using the R package vegan. Select genera are labeled based on their relevance to activated sludge functionality or distance from the origin. Most bacterial genera do not correlate strongly with any month, with notable exceptions such as *Cytophaga*, *Gordonia*, and *Aeromonas*.

Redundancy analysis revealed the organisms most strongly correlated with each month (Fig. 4); however, the majority of detected organisms cluster near the origin. *Cytophaga*, *Candidatus Cloacimonas,* and *Dietzia* were all correlated with the samples collected in February. *Methanosaeta* was the only genus detected from the domain *Archaea* and was also correlated with February. Of the classical functionally important genera for wastewater treatment, including *Tetrasphaera* and *Hyphomicrobium,* many were correlated with the month of December. Of the total variation captured by the RDA, 91% of it was explained by sampling date (Table S5).

## DISCUSSION

Throughout the sampling period, the microbial community included a large proportion of Proteobacteria, which is consistent with other metagenomic analyses of activated sludge environments (28, 29). In the context of wastewater, this phylum is composed of transient organisms primarily native to influent (e.g., *Acinetobacter* sp.) as well as organisms that are known to contribute to nutrient removal in activated sludge systems, such as members of the class Betaproteobacteria (30). Phylum Actinobacteria was also well-represented in this microbial community; however, fewer reads were classified to the family and genus level, which could be a result of database bias or potentially the presence of previously uncharacterized organisms without described marker genes available for the analysis. At the genus level, the most highly represented members across samples were *Thiomonas*, *Tetrasphaera*, *Afipia*, and *Hyphomicrobium. Tetrasphaera* and *Hyphomicrobium* are both of special interest in this environment because they perform key metabolic roles in nutrient removal, such as phosphorus accumulation and complete denitrification, respectively (31, 32).

The microbial community described by the recovered collection of quality MAGs differs from the results of the read-based analysis, particularly for the most abundant organisms. For example, no bins were classified as *Thiomonas* or *Hyphomicrobium*, despite these organisms being among the most abundant in the read-based analysis. However, there was some overlap between classifications made by the two approaches, including genera *Leucobacter, Dietzia, Gordonia, Cytophaga, Cloacimonas,* and *Brevundimonas*.

An additional difference between the organisms detected by the assembly-based approach and the read-based approach was the abundance of organisms from the phylum Bacteroidetes. Bacteroidetes are well-adapted to the human intestinal tract and therefore their presence in municipal wastewater is expected (33). The read-based analysis detected Bacteroidetes in consistently low abundance (<5%) (Fig. 1), primarily in the month of February, while the assembly-based analysis detected organisms from Bacteroidetes throughout the year and found that this phylum represented a quarter of the collection of recovered MAGs.

A noteworthy difference between the activated sludge microbial community in this study and those previously described in the literature is the presence of the phylum Actinobacteria in high abundance. Previous studies have found this phylum to account for less than 10% of the community (34 – 36), whereas, in this system, it accounted for approximately half of the community. The presence of Actinobacteria in high abundance in activated sludge has been linked to adverse outcomes in wastewater treatment, particularly in domestic wastewater (37). Although some members of this phyla are causative agents of operational challenges like sludge bulking and foaming (37 – 39), it is unclear what role the organisms of this phyla are playing in the system described in this study due to the classification being limited to the family level. The read-based analysis classified the majority of reads from Actinobacteria as belonging to the family Dermatophilaceae*,* which contains both genera that negatively and positively contribute to BNR. For example, this family includes *Kineosphaera* spp., an organism originally isolated from a deteriorated enhanced biological phosphorus removal (EBPR) activated sludge system, and putative glycogen-accumulating organisms (GAOs) (31, 40). GAOs are theorized to compete with phosphorus-accumulating organisms (PAOs) for resources in EBPR systems without contributing to phosphorus removal, thereby reducing the efficacy of these systems (41). However, GAOs have only been demonstrated to outcompete PAOs in lab-scale experiments and not in any full-scale WWTPs (42, 43). Additionally, other GAOs have been detected in large quantities in full-scale WWTPs with stable performance (44), suggesting the mere presence of these organisms is not sufficient to cause operational deficiencies. Additionally, the family Dermatophilaceae also contains *Candidatus Phosphoribacter* and *Candidatus Lutibacillus*, which are dominant PAOs in EBPR wastewater treatment plants worldwide (45). All quality MAGs as well as the majority of total MAGs that did not meet quality thresholds recovered from this family were classified as *Ca. Phosphoribacter*, which suggests that the high abundance of Actinobacteria in this environment is not inherently problematic.

Diversity, as represented by the Inverse Simpson index, varied significantly between the four dates when sampling occurred. Inverse Simpson values ranged from 4.4 to 7.8, with December consistently reporting the highest diversity values. April had the lowest microbial diversity as well as the lowest reported nutrient removal efficiencies of the 4 months sampled. Bioreactor operational difficulties such as foaming, bulking, or sporadic EBPR failure are often accompanied by the overgrowth of select taxa and as a result, reduced overall microbial diversity, which suggests there may be a link between diversity and bioreactor performance (46, 47).

Genes involved in denitrification were present in greater read abundance than those involved in nitrification. Given that the oxidation of ammonia and nitrite is a specialized trait that is only known to occur in select taxa, it is not surprising that the genes involved in this process would not be detected in equally high levels (48, 49). The abundance of the genes involved in denitrification was present in varying abundances depending on the enzyme, with NO_2_ reductase being the lowest (1.18 ± 0.69 copies per million reads), and NO_3_ reductase being the highest (36.99 ± 2.54 copies per million reads). This pattern is consistent with surveys of denitrification genes in other environments, including riparian areas and soils (50, 51). The comparatively high abundance of nitrate reductase was also reflected in the collection of recovered MAGs, where the majority of genomes included genes for either periplasmic nitrate reductase or respiratory nitrate reductase. Given the ability to completely reduce nitrate to nitrogen gas is not widespread among denitrifying organisms (52), the lack of MAGs encoding genes for all four denitrification enzymes is expected. Furthermore, it is possible that more sequencing data would resolve additional denitrification genes within the detected MAGs based on the results from Nonpareil (Fig. S1).

### Conclusion

Activated sludge is a dynamic system that relies on a complex, balanced microbial community. Here, read-based and assembly-based metagenomic analyses were employed to explore the changes in the activated sludge microbial community and its response to changes in temperature. Through MAG analysis, we were able to identify bacteria that were not detected in the read-based analysis and vice versa, demonstrating that both of these approaches can be used in parallel to better characterize and explore microbial communities. This activated sludge community was dominated by Proteobacteria with an unexpected abundance of Actinobacteria compared to other activated sludge systems. Additionally, genes involved in the denitrification pathway were distributed widely across different organisms (MAGs), while genes involved in nitrification were absent. Although shifts in the microbial community were observed across months, genes involved in nitrification and denitrification pathways remained stable, suggesting that the efficacy and robustness of this system rely on more than just the taxonomic composition of the microbial community. Particularly for denitrification, functionality in this system seems to be redundant among bacterial groups, and the relative contributions of these organisms could be better understood through transcriptional analyses. A deeper understanding of the organisms involved in biological nutrient removal and their contributions to the process is necessary to maintain WWTP systems with low failure rates and to promote the design of high-efficiency WWTPs.

## Data Availability

Sequencing data used in this study are available at the Sequence Read Archive (SRA) under study accession number PRJNA952735.

## SUPPLEMENTAL MATERIAL

Supplemental tables and figures spectrum.02832-23-s0001.pdf Figure S1 and Tables S1, S2, S4, and S5.

Table S3 spectrum.02832-23-s0002.xlsx Details about metagenome assembled genomes discussed in the paper.

ASM does not own the copyrights to Supplemental Material that may be linked to, or accessed through, an article. The authors have granted ASM a non-exclusive, world-wide license to publish the Supplemental Material files. Please contact the corresponding author directly for reuse.
