# Supplementary material for: Temporal metagenomic characterization of microbial community structure and nitrogen modification genes within an activated sludge bioreactor system
Source: Microbiol Spectr. 2023 Nov 29;12(1):e02832-23. doi: 10.1128/spectrum.02832-23 (PMC10783093; doi:10.1128/spectrum.02832-23)
Supplement: Supplemental tables and figures — Figure S1 and Tables S1, S2, S4, and S5. [file spectrum.02832-23-s0001.pdf]

Supplementary Figures and Tables

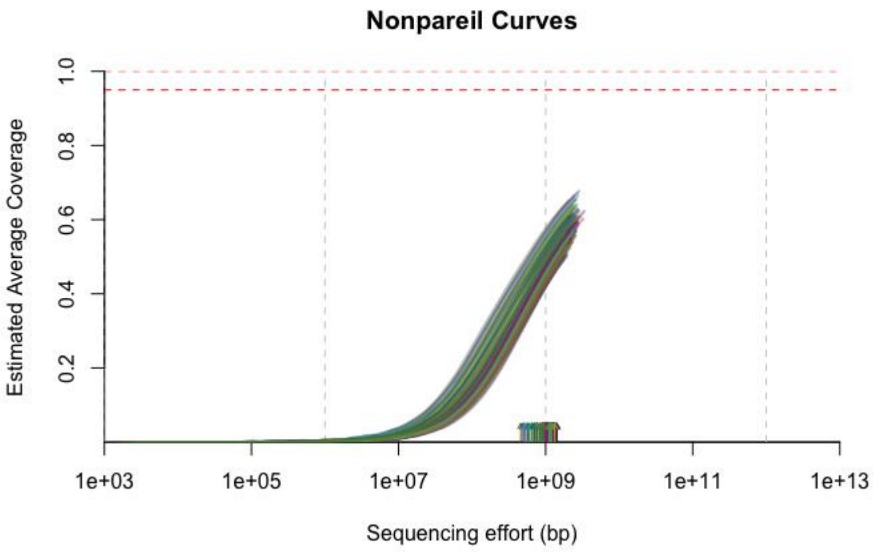

Figure S1: Nonpareil curves calculated for each metagenomic sample. Nonpareil estimates the extent of sequencing coverage.

Table S1. WWTP operational parameters during sampling dates. H_2O temp refers to the temperature of the influent pumped in from the city of Regina before it has undergone any treatment. The ambient temperature was obtained from Environment and Climate Change Canada and all other measurements were collected by personnel at the Regina WWTP.

Date	Ambient Temp	H_2O Temp	N: % Removal	P: % Removal
**19 DEC 2017**	-7.6 ºC	14.8 ºC	99.07	98.43
**13 FEB 2018**	-1.8 ºC	11.83 ºC	98.90	94.84
**17 APR 2018**	2.2 ºC	11.46 ºC	91.89	69.58
**10 JUL 2018**	28 ºC	18.5 ºC	98.98	85.46

Table S2. Sequence Statistics. Libraries were prepared and sequenced at Genome Quebec (Montréal, Québec).

Libraries were sequenced on three lanes of an Illumina HiSeq 4000, generating paired end, 150-bp reads.

Sample Name	Seq count (M)	% Duplicated Reads	% GC Content	Length
HI.4860.001.NS_Adaptor_10.FEB-C4_R1	19.4	3.8%	59.0%	150 bp
HI.4860.001.NS_Adaptor_10.FEB-C4_R2	19.4	3.2%	59.0%	150 bp
HI.4860.001.NS_Adaptor_11.FEB-C8_R1	20.1	3.3%	58.0%	150 bp
HI.4860.001.NS_Adaptor_11.FEB-C8_R2	20.1	2.9%	58.0%	150 bp
HI.4860.001.NS_Adaptor_12.APR-A1_R1	20.4	4.1%	59.0%	150 bp
HI.4860.001.NS_Adaptor_12.APR-A1_R2	20.4	3.4%	59.0%	150 bp
HI.4860.001.NS_Adaptor_13.APR-A2_R1	21.7	4.6%	60.0%	150 bp
HI.4860.001.NS_Adaptor_13.APR-A2_R2	21.7	4.0%	60.0%	150 bp
HI.4860.001.NS_Adaptor_14.APR-A4_R1	20.3	3.9%	59.0%	150 bp
HI.4860.001.NS_Adaptor_14.APR-A4_R2	20.3	3.3%	59.0%	150 bp
HI.4860.001.NS_Adaptor_15.APR-A8_R1	18.3	3.3%	57.0%	150 bp
HI.4860.001.NS_Adaptor_15.APR-A8_R2	18.3	2.9%	57.0%	150 bp
HI.4860.001.NS_Adaptor_16.APR-B1_R1	16.7	4.0%	61.0%	150 bp
HI.4860.001.NS_Adaptor_16.APR-B1_R2	16.7	3.3%	61.0%	150 bp
HI.4860.001.NS_Adaptor_17.APR-B2_R1	19.5	4.3%	60.0%	150 bp
HI.4860.001.NS_Adaptor_17.APR-B2_R2	19.5	3.7%	60.0%	150 bp
HI.4860.001.NS_Adaptor_18.APR-B4_R1	18.3	3.9%	60.0%	150 bp
HI.4860.001.NS_Adaptor_18.APR-B4_R2	18.3	3.4%	60.0%	150 bp
HI.4860.001.NS_Adaptor_19.APR-B8_R1	18.4	3.7%	59.0%	150 bp
HI.4860.001.NS_Adaptor_19.APR-B8_R2	18.4	3.1%	59.0%	150 bp
HI.4860.001.NS_Adaptor_4.FEB-B1_R1	18.3	3.2%	59.0%	150 bp
HI.4860.001.NS_Adaptor_4.FEB-B1_R2	18.3	2.7%	59.0%	150 bp
HI.4860.001.NS_Adaptor_5.FEB-B2_R1	18.3	3.4%	60.0%	150 bp
HI.4860.001.NS_Adaptor_5.FEB-B2_R2	18.3	2.9%	60.0%	150 bp
HI.4860.001.NS_Adaptor_6.FEB-B4_R1	21.9	3.9%	59.0%	150 bp
HI.4860.001.NS_Adaptor_6.FEB-B4_R2	21.9	3.2%	59.0%	150 bp
HI.4860.001.NS_Adaptor_7.FEB-B8_R1	21.6	3.5%	59.0%	150 bp
HI.4860.001.NS_Adaptor_7.FEB-B8_R2	21.6	3.1%	59.0%	150 bp
HI.4860.001.NS_Adaptor_8.FEB-C1_R1	22.6	3.6%	58.0%	150 bp
HI.4860.001.NS_Adaptor_8.FEB-C1_R2	22.6	3.0%	58.0%	150 bp
HI.4860.001.NS_Adaptor_9.FEB-C2_R1	21.1	3.2%	57.0%	150 bp
HI.4860.001.NS_Adaptor_9.FEB-C2_R2	21.1	2.8%	57.0%	150 bp
HI.4860.002.NS_Adaptor_1.JUL-A2_R1	25.6	3.2%	57.0%	150 bp
HI.4860.002.NS_Adaptor_1.JUL-A2_R2	25.6	2.6%	57.0%	150 bp
HI.4860.002.NS_Adaptor_10.JUL-C4_R1	20.2	3.0%	57.0%	150 bp
HI.4860.002.NS_Adaptor_10.JUL-C4_R2	20.2	2.4%	57.0%	150 bp
HI.4860.002.NS_Adaptor_11.JUL-C8_R1	16.5	2.7%	56.0%	150 bp
HI.4860.002.NS_Adaptor_11.JUL-C8_R2	16.5	2.1%	56.0%	150 bp
HI.4860.002.NS_Adaptor_2.JUL-A4_R1	15.5	2.4%	56.0%	150 bp
HI.4860.002.NS_Adaptor_2.JUL-A4_R2	15.5	1.8%	56.0%	150 bp
HI.4860.002.NS_Adaptor_20.APR-C1_R1	18.5	4.0%	59.0%	150 bp
HI.4860.002.NS_Adaptor_20.APR-C1_R2	18.5	3.3%	59.0%	150 bp

HI.4860.002.NS_Adaptor_21.APR-C2_R2	22.2	3.9%	60.0%	150 bp
HI.4860.002.NS_Adaptor_22.APR-C4_R1	19.9	4.0%	59.0%	150 bp
HI.4860.002.NS_Adaptor_22.APR-C4_R2	19.9	3.3%	59.0%	150 bp
HI.4860.002.NS_Adaptor_23.APR-C8_R1	16.3	3.3%	58.0%	150 bp
HI.4860.002.NS_Adaptor_23.APR-C8_R2	16.3	2.5%	58.0%	150 bp
HI.4860.002.NS_Adaptor_24.JUL-A1_R1	18.5	2.7%	56.0%	150 bp
HI.4860.002.NS_Adaptor_24.JUL-A1_R2	18.5	2.1%	56.0%	150 bp
HI.4860.002.NS_Adaptor_3.JUL-A8_R1	20.1	2.8%	57.0%	150 bp
HI.4860.002.NS_Adaptor_3.JUL-A8_R2	20.1	2.2%	57.0%	150 bp
HI.4860.002.NS_Adaptor_4.JUL-B1_R1	15.9	2.6%	57.0%	150 bp
HI.4860.002.NS_Adaptor_4.JUL-B1_R2	15.9	2.0%	57.0%	150 bp
HI.4860.002.NS_Adaptor_5.JUL-B2_R1	18.6	2.9%	57.0%	150 bp
HI.4860.002.NS_Adaptor_5.JUL-B2_R2	18.6	2.2%	57.0%	150 bp
HI.4860.002.NS_Adaptor_6.JUL-B4_R1	21.3	3.0%	57.0%	150 bp
HI.4860.002.NS_Adaptor_6.JUL-B4_R2	21.3	2.3%	57.0%	150 bp
HI.4860.002.NS_Adaptor_7.JUL-B8_R1	20.3	3.0%	58.0%	150 bp
HI.4860.002.NS_Adaptor_7.JUL-B8_R2	20.3	2.4%	58.0%	150 bp
HI.4860.002.NS_Adaptor_8.JUL-C1_R1	22.3	3.4%	58.0%	150 bp
HI.4860.002.NS_Adaptor_8.JUL-C1_R2	22.3	2.8%	58.0%	150 bp
HI.4860.002.NS_Adaptor_9.JUL-C2_R1	20.4	3.0%	57.0%	150 bp
HI.4860.002.NS_Adaptor_9.JUL-C2_R2	20.4	2.3%	57.0%	150 bp
HI.4860.008.NS_Adaptor_1.FEB-A2_R1	22.7	3.4%	58.0%	150 bp
HI.4860.008.NS_Adaptor_1.FEB-A2_R2	22.7	3.0%	58.0%	150 bp
HI.4860.008.NS_Adaptor_12.DEC-A1_R1	18.8	2.8%	59.0%	150 bp
HI.4860.008.NS_Adaptor_12.DEC-A1_R2	18.8	2.5%	59.0%	150 bp
HI.4860.008.NS_Adaptor_13.DEC-A2_R1	18.3	2.9%	60.0%	150 bp
HI.4860.008.NS_Adaptor_13.DEC-A2_R2	18.3	2.5%	60.0%	150 bp
HI.4860.008.NS_Adaptor_14.DEC-A4_R1	18.6	2.8%	59.0%	150 bp
HI.4860.008.NS_Adaptor_14.DEC-A4_R2	18.6	2.6%	59.0%	150 bp
HI.4860.008.NS_Adaptor_15.DEC-A8_R1	17.6	2.8%	60.0%	150 bp
HI.4860.008.NS_Adaptor_15.DEC-A8_R2	17.6	2.4%	60.0%	150 bp
HI.4860.008.NS_Adaptor_16.DEC-B1_R1	14.9	2.7%	61.0%	150 bp
HI.4860.008.NS_Adaptor_16.DEC-B1_R2	14.9	2.4%	61.0%	150 bp
HI.4860.008.NS_Adaptor_17.DEC-B2_R1	17.3	2.7%	59.0%	150 bp
HI.4860.008.NS_Adaptor_17.DEC-B2_R2	17.3	2.4%	59.0%	150 bp
HI.4860.008.NS_Adaptor_18.DEC-B4_R1	18.2	2.7%	60.0%	150 bp
HI.4860.008.NS_Adaptor_18.DEC-B4_R2	18.2	2.4%	60.0%	150 bp
HI.4860.008.NS_Adaptor_19.DEC-B8_R1	17.4	2.7%	59.0%	150 bp
HI.4860.008.NS_Adaptor_19.DEC-B8_R2	17.4	2.4%	59.0%	150 bp
HI.4860.008.NS_Adaptor_2.FEB-A4_R1	15.4	2.9%	59.0%	150 bp
HI.4860.008.NS_Adaptor_2.FEB-A4_R2	15.4	2.5%	59.0%	150 bp
HI.4860.008.NS_Adaptor_20.DEC-C1_R1	20.6	3.1%	60.0%	150 bp
HI.4860.008.NS_Adaptor_20.DEC-C1_R2	20.6	2.8%	60.0%	150 bp
HI.4860.008.NS_Adaptor_21.DEC-C2_R1	21.1	3.0%	58.0%	150 bp
HI.4860.008.NS_Adaptor_21.DEC-C2_R2	21.1	2.5%	58.0%	150 bp
HI.4860.008.NS_Adaptor_22.DEC-C4_R1	18.8	2.6%	58.0%	150 bp
HI.4860.008.NS_Adaptor_22.DEC-C4_R2	18.8	2.3%	58.0%	150 bp

HI.4860.008.NS_Adaptor_23.DEC-C8_R1	16.9	2.6%	59.0%	150 bp
HI.4860.008.NS_Adaptor_23.DEC-C8_R2	16.9	2.3%	59.0%	150 bp
HI.4860.008.NS_Adaptor_24.FEB-A1_R1	19.1	2.9%	56.0%	150 bp
HI.4860.008.NS_Adaptor_24.FEB-A1_R2	19.1	2.5%	56.0%	150 bp
HI.4860.008.NS_Adaptor_3.FEB-A8_R1	20.2	3.1%	58.0%	150 bp
HI.4860.008.NS_Adaptor_3.FEB-A8_R2	20.2	2.7%	58.0%	150 bp

Table S4. Hypothesis testing of generalized linear model for differences in sample diversity between months. The effect of bioreactor and oxic zone was insignificant and were not included in comparisons. Null hypothesis tested was that there are no differences between any months, the null hypothesis was rejected at level of $\alpha=0.05$.

Null hypotheses	Effect estimate	Standard error	Z ratio	P-value
DEC – FEB = 0	1.4216	0.311	4.576	<0.0001*
DEC – APR = 0	1.4348	0.31	4.623	<0.0001*
DEC – JUL = 0	1.3553	0.312	4.342	0.0001*
FEB – APR = 0	0.0132	0.274	0.048	1.0000
FEB – JUL = 0	-0.0663	0.276	-0.241	0.9951
APR – JUL = 0	-0.0796	0.275	-0.289	0.9916

Table S5. RDA effect sizes for full model and individual model terms. Redundancy analysis was performed at the genus level using the R package, vegan. RDA revealed that most of the variation in these samples could be explained by the date on which it was collected, indicating that bioreactor and zone had limited influence.

	Degrees of freedom	Variance	F value	Pr (>F)
Full model	8	0.035005	9.7297	0.001*
Bioreactor	2	0.001333	1.4817	0.138
Zone	3	0.001748	1.2956	0.186
Date	3	0.031925	23.6625	0.001*
Residual	39	0.017539		
